# Ancestry: How researchers use it and what they mean by it

**DOI:** 10.3389/fgene.2023.1044555

**Published:** 2023-01-23

**Authors:** Bege Dauda, Santiago J. Molina, Danielle S. Allen, Agustin Fuentes, Nayanika Ghosh, Madelyn Mauro, Benjamin M. Neale, Aaron Panofsky, Mashaal Sohail, Sarah R. Zhang, Anna C. F. Lewis

**Affiliations:** ^1^ Center for Global Genomics and Health Equity, University of Pennsylvania, Philadelphia, PA, United States; ^2^ Department of Sociology, Northwestern University, Evanston, IL, United States; ^3^ Edmond & Lily Safra Center for Ethics, Harvard University, Cambridge, MA, United States; ^4^ Department of Anthropology, Princeton University, Princeton, NJ, United States; ^5^ Department of the History of Science, Harvard University, Cambridge, MA, United States; ^6^ Broad Institute of Harvard and MIT, Cambridge, MA, United States; ^7^ Analytic and Translational Genetics Unit, Massachusetts General Hospital, Boston, MA, United States; ^8^ Center for Genomic Medicine, Massachusetts General Hospital, Boston, MA, United States; ^9^ Institute for Society & Genetics, University of California, Los Angeles, Los Angeles, CA, United States; ^10^ Department of Public Policy, University of California, Los Angeles, Los Angeles, CA, United States; ^11^ Department of Sociology, University of California, Los Angeles, Los Angeles, CA, United States; ^12^ Centro de Ciencias Genomicas (CCG), Universidad Nacional Autonoma de Mexico (UNAM), Cuernavaca, Morelos, Mexico; ^13^ University of California, Berkeley, Berkeley, CA, United States; ^14^ Division of Genetics, Department of Medicine, Brigham and Women’s Hospital, Boston, MA, United States

**Keywords:** population descriptors, ancestry, genetic ancestry, race, ethnicity, population labeling

## Abstract

**Background:** Ancestry is often viewed as a more objective and less objectionable population descriptor than race or ethnicity. Perhaps reflecting this, usage of the term “ancestry” is rapidly growing in genetics research, with ancestry groups referenced in many situations. The appropriate usage of population descriptors in genetics research is an ongoing source of debate. Sound normative guidance should rest on an empirical understanding of current usage; in the case of ancestry, questions about how researchers use the concept, and what they mean by it, remain unanswered.

**Methods:** Systematic literature analysis of 205 articles at least tangentially related to human health from diverse disciplines that use the concept of ancestry, and semi-structured interviews with 44 lead authors of some of those articles.

**Results:** Ancestry is relied on to structure research questions and key methodological approaches. Yet researchers struggle to define it, and/or offer diverse definitions. For some ancestry is a genetic concept, but for many—including geneticists—ancestry is only tangentially related to genetics. For some interviewees, ancestry is explicitly equated to ethnicity; for others it is explicitly distanced from it. Ancestry is operationalized using multiple data types (including genetic variation and self-reported identities), though for a large fraction of articles (26%) it is impossible to tell which data types were used. Across the literature and interviews there is no consistent understanding of how ancestry relates to genetic concepts (including genetic ancestry and population structure), nor how these genetic concepts relate to each other. Beyond this conceptual confusion, practices related to summarizing patterns of genetic variation often rest on uninterrogated conventions. Continental labels are by far the most common type of label applied to ancestry groups. We observed many instances of slippage between reference to ancestry groups and racial groups.

**Conclusion:** Ancestry is in practice a highly ambiguous concept, and far from an objective counterpart to race or ethnicity. It is not uniquely a “biological” construct, and it does not represent a “safe haven” for researchers seeking to avoid evoking race or ethnicity in their work. Distinguishing genetic ancestry from ancestry more broadly will be a necessary part of providing conceptual clarity.

## Introduction

The use of population descriptors is currently under the spotlight, both in genetics research specifically and across biomedical research more broadly (National Academies 2021; Vyas, Eisenstein, and Jones 2021; Khan et al., 2022). Social scientists have studied how race and ethnicity are used, but have paid much less attention to ancestry. Understanding how researchers conceptualize and use ancestry matters—both to genetics and to biomedicine more broadly—for several reasons. First, it is a key concept drawn upon in decisions about who we study and why (Popejoy and Fullerton 2016; Bentley, Callier, and Rotimi 2017). Second, because it plays a key role in making sure methodologies yield robust and replicable results (Martin et al., 2019; Peterson et al., 2019). Third, because further reliance on genetic ancestry is part of the proposed solution to the use of race in biomedicine (Borrell et al., 2021; Oni-Orisan et al., 2021). Fourth, decisions made in research directly impact translational work, and ultimately medical practice (Popejoy et al., 2018). And finally, understanding this concept is important because it is a key frame offered for understanding biological differences between groups of humans—including those that could be driving race-based health disparities (Batai, Hooker, and Kittles 2021). This is an ethically fraught topic with a long and unpleasant history (Reardon 2005; Roberts 2011; Bliss 2020a). The stakes are hence high to ensure that concepts originating in genetics do not result in repetition of past atrocities stemming from the categorization of humans into a small number of biological types (Mathieson and Scally 2020; Lewis et al., 2022).

A better understanding of how researchers use ancestry can also help provide raw material for normative recommendations about how the concept should be used. The National Academies of Science, Engineering and Medicine (NASEM) are currently convening a taskforce on the appropriate usage of race, ethnicity and ancestry in genetics research (National Academies 2021). The appropriate usage of race, ethnicity, and ancestry is not a new topic: a scoping review found over 100 articles offering relevant normative guidance published since 2000 (Mauro et al., 2022). A consistent theme in these recommendations has been the need for transparency by researchers, including why they are using population categories, and how any population categories used are defined (Mauro et al., 2022). The use of race and ethnicity have been the main focus of these normative debates to date, with ancestry receiving relatively little scrutiny. For example, guidance from the American Medical Association has solely focused on race and ethnicity (Flanagin et al., 2021). It has been explicitly suggested that ancestry is the least controversial of the population descriptors (Lee, Mountain, and Koenig 2001), and this assumption seems to drive much of the move away from race and ethnicity categories. It is also seen as the most objective classifier. Pointing out how race and ethnicity categories are broad, imprecise, and ambiguous, Borrell et al. write, “In contrast, ancestry is a fixed characteristic of the genome” (Borrell et al., 2021). Recent content analysis of research articles published in the *American Journal of Human Genetics* has shown that the term “ancestry” is increasingly used in genetics research (Byeon et al., 2021). As Wagner et al. have argued, the increasing focus on ancestry as a way to “frame human difference” should be a motivating factor to bring more attention to its use (Wagner et al., 2017).

There have been empirical insights into researchers’ use of ancestry. Articles using ancestry (compared to race or ethnicity) were the least likely to provide a rationale for its use (Ali-Khan et al., 2011). Interviews with health researchers have demonstrated the degree of confusion amongst researchers about the interrelationships between race and ethnicity and genetic differences between populations (Baer et al., 2013). Drawing on content analysis of articles published in *Nature Genetics* and interviews, Panofsky and Bliss explore geneticists’ use of population labels, demonstrating the increasing use of continental labels, which they argue are fundamentally ambiguous because they “blur racial and geographic understandings of population difference” (Panofsky and Bliss 2017). In an ethnography of geneticists’ use of principal components analysis (PCA) to capture genetic ancestry, Fujimura and Rajagopalan argue that while there are opportunities to update how the field thinks about human biological difference, race and ethnicity nonetheless enter into the concept of ancestry (Fujimura and Rajagopalan 2011). Focusing on biomedical articles using the terms “black”, “African” and “African American” Duello et al. find that most studies do not give a rationale for their focus on these populations, and conclude “we infer the authors of these studies believe African ancestry denotes a biological ‘race’ of people of common descent who share DNA unique from the rest of mankind” (Duello et al., 2021).

Researchers across multiple fields employ the concept of ancestry; it is not straightforwardly a concept that is “owned” by genetics. The concept of ancestry is often employed in everyday conversation, carrying sociocultural implications outside of its usage in genetics and health research. This use across contexts gives many opportunities for miscommunication about what ancestry is and is not, and what we can learn about or from it. Genetics has a special role in our understanding of ancestry, but, as mentioned above, this concept then diffuses out to translational research, to the practice of medicine, and to popular conceptions about the human family tree.

In this study, we employ a mixed methodology—a systematic literature analysis and semi-structured interviews—to offer a comprehensive examination of how ancestry is used by researchers. Because diverse domains use the concept of ancestry, and because they are of mutual relevance to each other, we include research from multiple disciplines. We seek to answer five questions about researchers’ use of ancestry. First, what types of research use ancestry? Second, when and why does ancestry enter the research process, i.e., what are the use cases for the concept? Third, what does ancestry mean to researchers, i.e., what definitions do researchers offer for the concept? Fourth, how is ancestry operationalized, i.e., how is this abstract concept made into a measurable observation? And finally, what types of population labels are used for ancestry categories? Answers to the first two questions help indicate just how important the concept of ancestry is in structuring both research questions and methodologies; answers to the remaining questions shed light on whether ancestry as currently conceptualized and operationalized can bear this heavy weight.

## Materials and methods

### Study design

This study employed two different methodologies to understand the use of ancestry by researchers: a systematic literature analysis and semi-structured interviews. The systematic literature analysis was performed on an original dataset composed of a sample of research articles in the population sciences. We designed this corpus of original research articles to capture as much of the diversity of the ways that ancestry is currently used by researchers as possible, in terms of divergent research questions and methodologies across disciplines. Given the motivation of our work to inform the use of ancestry and genetic ancestry across the biomedical sciences, we constrained the articles in our corpus to have at least some tangential relevance to human health. The study also aimed at diversity in terms of publication journals such that not only articles published in high impact factor journals were selected for the study, as this might represent a selection bias towards particular types of studies (e.g., Large N). We also identified a subset of these articles as engaging with the concept of ancestry particularly closely, and invited the first and last authors of these articles to participate in a semi-structured interview. This multi-method approach was designed to allow us to develop a robust understanding of researchers’ use of ancestry, with the systematic literature analysis revealing patterns of usage, and the interviews allowing us to understand why we observed these patterns. This strategy yielded 205 articles and 44 interviewees.

### Article inclusion strategy

All searches were based on Web Of Science (WOS) and conducted in February 2021. We restricted all searches to articles that contained “ancestry” in the title, abstract, or keywords, and then deployed two search strategies. First, we restricted articles to those concerning certain phenotypes, published from 2019 on. This focus on phenotypes ensured that we obtained diversity along other dimensions that we cared about, specifically research methodology. We chose a range of different types of conditions, all of which (like most health conditions) present known health disparities: COVID-19, prostate cancer, chronic kidney disease, and schizophrenia. These articles were filtered to just retain original research articles where the phenotype of interest was a central explanandum of the article. Second, additional searches were conducted in order to obtain a sufficient number of anthropology, social science, and public health articles. The start year was adjusted to ensure an adequate sample size; this meant starting in 2010 for sociology (extending back to 2010 was necessary to achieve sufficient articles to analyze), 2015 for anthropology, and 2020 for public health. For the anthropology and sociology searches, we also required that the term “health” appear in the title, abstract, or keywords. The results from all three searches were then filtered to retain articles that had either a connection to “health” broadly defined, or to human evolution, or to the characterization of human populations. The number of articles from each of the searches before and after filtering are given in Supplementary Table S1. The search strings and details of filtering are given in the Supplementary Material. The complete list of included articles is included as a Supplementary Material.

The search results were exported, and the PDFs of the articles and their Supplementary Information were downloaded. We used the abstract, journal, and affiliations of first and last authors to assign a primary subfield and field to each article (fields were anthropology, biology, medicine, public health, and sociology, see Supplementary Material for methodology details). The number of articles per field is given in Table 1, and per subfield in Supplementary Table S2. We also assigned a Country/Region to an article based on the Country/Region of the first author’s primary affiliation, see Table 1.

**TABLE 1:** Field and Country/Region of first author’s primary affiliation of the articles in our corpus. The countries within the “Others” category are Australia, Canada, New Zealand, Jamaica and Suriname. The US was not aggregated into a region because of the country’s dominance in the discourse around population descriptors in research, likely due to the historic antecedents on the use of race in the country.

		Number of articles (*N* = 205)
Field	Anthropology	20
	Biology	54
	Medicine	65
	Public Health	55
	Sociology	11
Country/Region	Africa	9
	Asia	20
	Europe	35
	Latin America	15
	U.S.	101
	Others	25

### Interviewee recruitment and interview guide

Based on reading the articles, we identified 97 that engaged with the concept of ancestry most closely. This was typically because they either used ancestry to frame or motivate their research question, because ancestry was evoked centrally in their methodology, or the term “ancestry” frequently occurred in the text. In order to ensure we heard from researchers at multiple career stages, we invited the first and last authors of these articles to participate. In a small handful of cases, interviewees recommended we contact a middle author or other close collaborator to interview. This yielded 190 names, 166 of which we found emails for. Of these 166, we interviewed 44 (27% response rate). The majority (29) were based in the United States, with 7 based in Europe, 6 in Central and South America, one in Canada, and one in India. The interviewees were assigned a subfield based on their training as inferred through their professional biographies, publication record, and in conversation during the interview. We achieved disciplinary diversity within our interviewees: 7 from anthropology, 14 from biology, 11 from medicine, 6 from public health, and 6 from sociology.

In the interviews, we utilized the article by virtue of which the participant was recruited to probe in greater depth how they use and think about ancestry. The semi-structured interview guide covered five areas: their background and the focus of their work; the justifications for their choices related to ancestry, and how they understood the limitations of these; conceptual questions, including “What does ancestry mean to you?’’; publishing and the mechanisms of funding; and their views on the status of the field.

Interviews were 1 hour long, and conducted by two interviewers, one with a biology background (AL) and one sociologist (SM). The interviews were conducted on a video conferencing platform, recorded, and auto-transcribed. These transcripts were then updated based on the recordings. Identifying information was removed. The interview study was deemed exempt by the Harvard University-Area Committee on the Use of Human Subjects (protocol ID IRB21-0496).

### Data analysis

The documents—the full text of the 205 articles and the 44 anonymized transcripts—were first coded. This involves identifying, highlighting, and annotating the appropriate sections of text corresponding to a set of codes. Around 3,000 sections of text from the articles were tagged with 27 codes, and about 2,300 to 34 codes from the interview transcripts. The list of codes covered are given in Supplementary Tables S3, S4, and further details of the development of these lists of codes and details of the coding process are given in the Supplementary Material.

On a code-by-code basis we then analyzed the sections of coded text for emergent themes. In addition to this qualitative analysis, we established two features of the articles which enable quantitative presentation of results. First, for those articles that operationalize ancestry, we coded the data type(s) they use to do so. Second, we categorized the type of population labels used in the articles. We used the types of population labels as previously analyzed in Panofsky and Bliss (Panofsky and Bliss 2017), with some minor adaptations: see Supplementary Table S5 for these types, with examples. We stress that our corpus of articles is not representative of any clearly defined set of literature, and hence that our results do not generalize to all population sciences. Instead this curated dataset is enriched to identify salient variables that shape researchers’ conceptions of ancestry and to describe a wide variety of uses of the concept in scientific work.

## Results

### What types of research use ancestry?

The 205 articles in our corpus had diverse research aims, which we group into five categories, giving examples.

The most commonly represented category was articles aiming to understand traits and outcomes. This includes identifying genetic variation linked to traits (often but not always *via* Genome Wide Association Studies (GWAS) (e.g., (Legge et al., 2019; Lin et al., 2019; Du et al., 2020))), identifying causal influences on a trait using Mendelian Randomization [e.g., (Jordan et al., 2019; Howe et al., 2020)], identifying the interplay between genetic and environmental factors [e.g., (X. Chen et al., 2019; Ding et al., 2020)], understanding the impact of structural determinants of health [e.g., (Thayer et al., 2017; Whaley 2020)], understanding the molecular mechanisms that contribute to a trait/health outcome [e.g., (Emami et al., 2019; Gohlke et al., 2019)], and controlling for genetics in understanding social traits (Boardman et al., 2010).

A second set of articles, and the second most represented category, aimed at understanding between-group differences in traits/outcomes. Some of these articles compare traits/outcomes between those of different population categories [e.g., (Weitz, Garruto, and Chin 2016; Wong et al., 2019)] or those with different percentages of a particular ancestry category [e.g., (Grizzle et al., 2019; Fritz et al., 2020)]. Some articles compare trait-associated genetic variation between ancestry groups [e.g., (Koga et al., 2020; Liu et al., 2020)]. Some of these articles explicitly couch their efforts in terms of understanding health disparities [e.g., (Boulter et al., 2015; Marden et al., 2016)].

A third set of articles focuses on understanding genetic structure. These articles aimed to describe and infer population history, to understand evolutionary processes [e.g., (Macholdt et al., 2015)], and gain insight into how present-day genetic diversity is shaped [e.g., (Leishangthem et al., 2020; Zhao et al., 2020)].

A fourth set of articles aimed to understand social identities. This includes understanding how ancestry and genetic ancestry relate to categorical frameworks used in the present such as race [e.g., (Liebler 2016; Paredes 2017)], and understanding what factors influence these social identities (Hunley et al., 2017). It also involves trying to gain insight into the life histories and lived experiences of those who lived in the past, particularly enslaved individuals [e.g., (Wasterlain, Costa, and Ferreira 2018; Fleskes et al., 2021)].

A final set of articles evoking ancestry were aimed at directly improving the provision of healthcare. This includes: improving patient/participant engagement for example by understanding the views of those of diverse ancestries (Menzies et al., 2020; Saad et al., 2020); developing clinical tools for example by consideration of incorporation of genetic ancestry as a variable [e.g., (Haas Pizarro et al., 2020; Canter et al., 2019)]; studying the impact of genetic testing, for example by reporting the diagnosis rate by ancestry group (Groopman, 2019); and enabling quality control, for example by comparing genetically inferred ancestry to self-reported data for cell lines (Hooker et al., 2019).

Some articles conducted research that spanned these categories. For example, many of the articles aimed at understanding traits (e.g., a GWAS) additionally include a between-group comparison (e.g., comparing the frequency of an identified variant across ancestry groups). We observed that many articles do not clearly lay out their aims, with the relationship between research question and research motivation somewhat diffuse.

### How does ancestry enter the research process?

Ancestry can enter the research process at multiple stages, representing different use cases for the concept.

As seen in the previous section, ancestry can be used to frame the research question when the focus is on the relevance of ancestry to a trait or outcome. The motivation for this is the assumption that ancestry reflects distinctive patterns of genetic difference, and that using it as a key variable will help identify whether genetic factors could be contributing to differences in incidence and prevalence rates of disease, or differences in traits, between groups [e.g., (Yuan et al., 2020)]. This can be couched explicitly in terms of understanding whether genetics plays a role in health disparities [e.g., (Apprey et al., 2019)].

Ancestry is also used to state the research question when it is seen as defining the population of interest. Two justifications for choice of population of interest were particularly common in our data. First, the lack of pre-existing research in that population, either noting the under-representation of those of certain ancestries in research generally, or the absence of a particular type of study in a particular population. To further strengthen the justification for using the named ancestral group in the study, some articles point to the consequences of not doing this work, for example to “*exacerbate existing health disparities”* (Harlemon et al., 2020). The second common justification given was the high prevalence of a phenotypic trait in that ancestry group/population.

Ancestry can also enter at the analysis stage. A notable example is to account for confounding in a GWAS, either in identifying suitable controls in a case-control study, or to account for population structure using principal components. While most use “ancestry” language explicitly to describe this process, some do not [e.g., (Ohi et al., 2020)], or circumscribe this use more carefully as “heterogeneity that is correlated with ancestry” (Franceschini and Morris 2020). Another example where ancestry is treated as something to be “controlled for” is in controlling for the influence of genetics when understanding a trait (Boardman et al., 2010), to help better understand the influence of other variables on that trait. Another example is in admixture mapping, which explicitly models aspects of genetic structure, using a process often referred to as local ancestry inference, in order to decrease bias in the estimates of the effect size of genotype-phenotype correlations [e.g., (Du et al., 2020)].

Finally, ancestry is evoked at the reporting stage of the research process, both in the statement of results and of the conclusions that follow from them [e.g., (Yoshikawa et al., 2020; Darst et al., 2020)]. This is true even when ancestry does not explicitly enter the research aim, for example in statements of how the results may or may not generalize to different populations (Tonon et al., 2019; Brhane et al., 2020).

### What does ancestry mean to researchers?

We’ve seen that ancestry is used to frame research questions and as a core part of research methodologies. What do researchers mean by it? Many researchers—all of whom were selected to interview because their work closely engaged with the concept of ancestry—struggled to answer the question “What does ancestry mean to you?’’. While the majority did offer definitions, often after pauses, some were not able to: “Yeah it is hard, I do not know in fact.” We observed that interviewees who were the most engaged in the concept were the most uncertain about it, and/or who gave the most expansive, multipart definitions. An example of the former is an interviewee who remarked that defining ancestry was “like trying to catch smoke”. An example of the latter was an interviewee who emphasized that the more one thinks about the concept, the more expansive the conceptualization becomes: “it is not a simple (question), to answer, because there are of course different ways of thinking about it… each project, to some extent, helps you to rethink what (ancestry) means”. Consistent with ancestry being a concept from everyday life, many interviewees drew from their own personal stories in the answers that they gave.

We identified two dimensions along which answers differed. The first is what ancestry is a property of: DNA; the individual; their family/kin; a population. The second is the criteria by which ancestry is shared: geographical origin; genealogical connections; culture; biology. Examples are given in Table 2. The qualitative methodology we employed is not suitable to quantify the answers we received, but we note that the answers we received were well distributed along both of these dimensions. Of note, geneticists did not all just give definitions in terms of genetic information. The “culture” category encompasses shared ethnicity, but also more specifically, shared narrative. For example, one interviewee described ancestry as “intergenerational transmission of who I am, what my family is, what we do, who we are.”

**TABLE 2:** Categorizing the definitions of ancestry offered by researchers whose work closely engages the concept by a) what ancestry is a property of, and b) the criteria by which individuals share ancestry.

	Geographical origin	Genealogical connections	Culture	Biology
DNA	“The geographic origin of genetic variation”	“The genetic information that we inherit from our ancestors”		
Individual	“Direct roots... those who may have been born, lived” (in a particular place)		“What people themselves say when we collect samples, and we asked them… what ethno-linguistic group they belong to”	“Ancestry is related to your biological background… your biological roots”
Family/Kin	“Where your more recent ancestors came from geographically”	“We know it as genealogy”	“Full family tree.... not only what country but what’s their role in the country, and how does that relate to my identity”	
Population	“The history of the distribution of populations.”	“It’s just a bunch of people more sharing a common ancestor than another group of people.”		“Population differences in genotype that occurred over long periods of time, based on human migration”

Many individuals gave multipart answers spanning different cells in the matrix represented by Table 2. For example, “there’s two aspects to it, one sort of the social kind of ancestry that people know about, that they’re talking about, and the other one is then what genetics actually shows.” Or, “Mostly genetic heritage, I mean you know literal inheritance… and something more like culture or religion or ethnicity or identity or family or community or group”.

Some interviewees were keen to emphasize the distinction of ancestry from race and/or ethnicity. For example, “race is a self identified construct and ancestry is biology”, or “it is very far from concepts like, race or ethnicity or something like that.” But others directly related the concepts, for example “African American ancestry - that racial group that, you know, would essentially have common genotypic and phenotypic characteristics.” In some cases there was a direct conflation which was masked by language, “Previously I used ethnicity. But then my mentor told me that nowadays people use ancestry.’’, or as a term to be used “instead of race, because we cannot use, like ‘mixed race’ because… the connotation is not good.”

### How is ancestry operationalized?

Before it is used, ancestry first has to be operationalized, i.e. a process has to be defined to ascribe an ancestry to individuals.

Out of the 205 articles examined, 56 (27%) did not operationalize ancestry in their methodology or analysis. Many articles use the word “ancestry” haphazardly and interchange the term with other population descriptors. For example, an article states “More than half of our patients are from African ancestry” (Arleo et al., 2021), but uses race groups throughout. In these cases, the authors seem to be grasping for a term that is suitably inclusive to cover a range of type of variation, or to use a word viewed as unobjectionable (in comparison to race or ethnicity), or to use a term that sounds more objective. Some use the term specifically to draw attention to the lack of data outside of European ancestry populations (Guan et al., 2020). The term “ancestry” appears as a label for ethnic groups in two main cases: “indigenous ancestry” (Marziali et al., 2021) and “mixed ancestry” (Hill et al., 2020). Finally, some articles only contained mention of “ancestry” in the keywords (and not in the main body of the text), using the “ancestry group” MESH terms (Dina 2022).

Of the remaining 149 articles that did operationalize ancestry, for 39 (26%) of these we could not determine what type of information was used in this operationalization (e.g., self report, geographic, genetic). For example, when articles simply refer to “men of African ancestry” (Lam et al., 2019; Walavalkar et al., 2020). In some of these cases, it seems that the choice of population descriptor was not carefully made. For example, one article describes its sample as racially diverse, but demonstrates this using *European ancestry*, *African ancestry* and *other* categories (Gur et al., 2019). In another paper, the authors refer to *European*, *African* and *Mixed ancestries* in some places, but in others use *Mixed ancestry*, *Black African*, and *Caucasian* (Passchier et al., 2020). Some researchers use ancestry and ethnicity synonymously (Franceschini and Morris 2020).

Of the 110 articles where it was possible to tell what type of data was used to operationalize ancestry, 72 (65%) used genetic information to do so, 53 (49%) used non-genetic data, and 15 (14%) used both genetic and non-genetic data, see Table 3. Of the 38 articles that used exclusively non-genetic data to operationalize ancestry, 14 were genetics papers. The non-genetic data types used to operationalize ancestry were: self-reported information, geographical information, language spoken, surnames, dental morphology, and sometimes the intersection of more than one of these sources. Eleven articles (10%) used an intersection of genetic and non-genetic data types. Eight articles (8%) operationalized ancestry in more than one way in the same paper.

**TABLE 3:** Types of data used to operationalize ancestry in articles

Type of data used to operationalize ancestry		N (%)	N (%)
Genetic			57 (52)
	Just genetic	53 (48)	
	Multiple operationalizations: genetic and not specified	4 (4)	
Non-genetic			38 (35)
	Dental morphology	1 (1)	
	Geographic	9 (8)	
	Language	1 (1)	
	Self report	20 (18)	
	Surname	4 (4)	
	Intersect of non-genetic data types	3 (3)	
Both genetic and non-genetic			15 (14)
	Intersect of genetic and self report	11 (10)	
	Multiple operationalizations: genetic and non-genetic	4 (4)	
TOTAL		110 (100)	110 (100)

For those articles for which it was possible to determine the type of information used to operationalize ancestry, this information was often hard to find. For example, in Harrison et al., the main text referred to their Supplementary Material, which in turn pointed to a preprint. In the Supplementary Material of the preprint the details of how ancestry was operationalized are given (Harrison et al., 2020).

Most of the articles that used self-identified information to operationalize ancestry are consistent with participants being asked to report their race and/or ethnicity rather than their ancestry. Suggestive evidence of this includes interchangeable use of ethnicity and ancestry, reference to “racial ancestry” (Gendy et al., 2019; Kaur et al., 2019), and demonstration of “racial diversity” using ancestry categories (Dupont et al., 2020). A notable source of data where individuals are actually asked to self-report their ancestry is the American Community Survey (ACS), administered by the US Census Bureau. It currently asks, “What is your ancestry or ethnic origin? (For example: Italian, Jamaican, African American, Cambodian, Cape Verdean, Norwegian, Dominican, French Canadian, Haitian, Korean, Lebanese, Polish, Nigerian, and so on.)". The ACS was used by six articles in our corpus: four from public health and two from sociology.

There was also diversity in how geographic data was used to operationalize ancestry: birth country of parents (Hamilton et al., 2018; Yamasato et al., 2020); birth country of paternal grandfather (Groeger et al., 2017); all of mother, father, and four grandparents born in a particular geographical area (Martínez-Magaña et al., 2019); or, no clarity beyond the mention of a country (Chen et al., 2019).

Ancestry was also operationalized using surnames, including: inferred place of origin of the surname of the individual (Bakhtiari 2020); whether an individual’s two surnames (one inherited from each parent) in Mexico were deemed Mayan (Azcorra et al., 2016); the fraction of the surnames of each of an individual’s two parents deemed Andean in Peru (Pomeroy et al., 2015).

The articles in our corpus that used genetic data to operationalize ancestry used a variety of methodologies to do so. Some used analysis of Ancestry Informative Markers (AIMs), which are genetic variants specifically picked because they have very large frequency differences in different groups, where the groups of interest are chosen during design of the AIMs. The majority of articles used standard genotype chips and one of a small handful of methodologies. One common method is Principal Component Analysis (PCA), a dimension reduction technique that defines a space in which the first dimension (PC) captures the most variance in the inputted data, the second the next most, etc. Another common method is a version of the STRUCTURE/ADMIXTURE algorithms, which assign each individual a percentage of ancestry in different variation distribution clusters referred to as populations. These algorithms can be run in an unsupervised fashion, where the user provides only the number of populations (referred to as the “k” parameter). It can also be run in a supervised fashion, where the user defines the populations by providing reference genetic data for each. Of course, the labels for these populations are previously defined using some other form of data, typically geographic or self-identified. Methodologies other than these two were sometimes used, notably Multidimensional Scaling e.g., (Maciukiewicz et al., 2019).

As previously mentioned, many articles use PCA in their analysis; most of these refer to an individual’s location in Principal Component space as their ancestry, though exceptions include referring to this as ethnicity (Yoshikawa et al., 2020). In interviews, amongst those closest to population genetics, the relationship between PCs, population structure, and genetic ancestry was couched in diverse ways: ancestry as the “interpretation” of population structure (viewed as PCs) or as “arising out of” population structure; ancestry as “a lot more than” population structure, (interpreted as PCs); PCs as “indicators” of ancestry. Some articles view use of PCs as a quality control step to “confirm” participants’ self-reported ancestry (Maciukiewicz et al., 2019) or ethnicity (Yoshikawa et al., 2020).

When PCs were used to define a population, this was typically done through drawing ellipses (“bubbles”) in PC space. Our interviewees reported that this was mostly either an “eyeball” process, or simply following a rule of thumb because that is what another paper had done. When PCs were used to control for confounding, the number used to do so varied widely, and, when asked, interviewees explained they were either choosing a number based on what a previous paper had used, or followed a rule of thumb (such as the “elbow rule”, whereby visual inspection for a dropoff in the amount of variance explained with additional PCs is used to define a cutoff (Cattell 1966)). Few were able to further justify why this was appropriate. While most of our interviewees used PCs somewhat blindly, others drew attention to issues with the use of PCs, including that they depend entirely on the data inputted, that they are hard to interpret and it is not clear what they are actually picking up on, and that they can pick up on non “real” population structure, due to relatedness or QC issues. One interviewee pointed out that many databases are making PCs for their data available, increasingly enabling the “off the shelf” use of this way to operationalize ancestry.

With the use of statistical software packages such as STRUCTURE/ADMIXTURE, which characterize ancestry of an individual in terms of their percentage similarity with different populations, many interviewees showed a greater awareness of the ways it could be arbitrary, reflecting the fact that choices must be made to run it (either by providing reference data of the populations of interest, or by providing the k parameter). For example, one interviewee described the extent to which interesting observations could be made about their data as k varied (e.g., 3 vs. 5 populations using the same data). Some researchers, again those closest to population genetics, offered additional cautions about the use of this methodology to operationalize ancestry, including that it is very easy to over interpret the results, that newer admixture can be confused with identity by descent, and that there never have been “pure” populations. This last observation refers to the fact that the underlying population theoretic model motivating the design of STRUCTURE/ADMIXTURE models an individual as deriving proportions of ancestry from well-defined ancestral populations, but any such populations are themselves mixtures of other populations.

Examples of the “Genetic Intersect Self Report” operationalizations are found in articles that used the United Kingdom Biobank “White British” data (Harrison et al., 2020) (Kolin et al., 2020), which uses a “bubble” in genetic principal component space combined with a filter on an ethnicity category, and others with self-reported information that was further analyzed using multi-dimensional scaling (MDS) in PLINK (Avinum et al., 2020).

We observed inconsistent use of terminology to describe ancestry operationalized using genetic data. Articles were very mixed about whether they referred to this as “genetic ancestry”, with about equal numbers systematically using “genetic ancestry” or using just “ancestry”, and with many others using a mixture of these two. Other terms in use include “population ancestry”, “genomic ancestry”, “ancestral population structure’’, and “DNA ancestry”. Additionally, some articles distinguished global from local ancestry, but meant different things by this distinction. For some, “global ancestry” means the percentage of ancestral populations inferred by programs like ADMIXTURE (typically, but not always, continental ancestral populations) whereas “local ancestry” involves assigning population labels to sections of chromosome (others used “chromosomal ancestry” for this (Chen et al., 2020)). For other researchers, “global ancestry” means continental ancestry, and “local ancestry” indicates finer-resolution categories, e.g., country-level ancestry.

We observed a similar lack of clarity around how the term “population” is used by researchers. For some, a population is a model from population genetics implying random mating. For others, drawing from statistics, data from a sample should be chosen such that it is representative of a population. But for most, usage was more diffuse, implying simply a group of people with something (anything) in common, or as one interviewee put it, simply “large N” (where N is the sample size).

We investigated how the data type used to operationalize ancestry varies across fields of study: anthropology, biology, medicine, public health, and sociology, see Table 4. All fields of study used both genetic and other data types to operationalize ancestry. Genetic data is the most common data type used in biology, (22 operationalizations, 45% of those in biology), medicine (22, 40%), and public health (12, 39%). Of the 157 operationalizations, 119 appeared in articles that used genetic data, and it was not the case that they exclusively used genetic data to do so. Only 61 (51%) used exclusively genetic information, 11 (9%) used genetic and non-genetic information, 18 (15%) used exclusively non-genetic information (both geographic and self report), and 29 (24%) did not specify which type of data was used. The rates of not specifying what data were used to operationalize ancestry were particularly high in biology (15, 31%) and medicine (19, 35%).

**TABLE 4:** Types of data used to operationalize ancestry by articles in different fields. Note that the 8 articles that operationalized ancestry in more than one way appear more than once in this table.

Data type	Primary field					
	Anthropology N (%)	Biology N (%)	Medicine N (%)	Public health N (%)	Sociology N (%)	Total N (%)
Genetic	4 (29)	22 (45)	22 (40)	12 (39)	1 (13)	61 (39)
Genetic Intersect Non-genetic	−	4 (8)	3 (5)	4 (13)	−	11 (7)
Non-genetic	8 (57)	8 (16)	11 (20)	9 (29)	6 (75)	42 (27)
Not specified	2 (14)	15 (31)	19 (35)	6 (19)	1 (13)	43 (27)
Total	14 (100)	49 (100)	55 (100)	31 (100)	8 (100)	157 (100)

Throughout our analysis of the operationalization of ancestry we observed many sources of conflation between ancestry and race and ethnicity. As mentioned, it seems likely that many of the participants who were listed as self-reporting their ancestry actually self-reported their race or ethnicity. This is likely also true for the articles where it was not specified what type of data they used to operationalize ancestry. Other articles referred to their ancestry categories inferred from genetic data as ethnicities (Bani-Fatemi et al., 2019). One article refers to “race assigning” using either self report or tertile of genetically inferred West African ancestry (Gohlke et al., 2019).

### What types of population labels are used for ancestry categories?

Just as there is diversity in what type of data is used to operationalize ancestry, there is also diversity in the types of population labels used to describe the resulting categories (see Table 5). Continental ancestry is the most used population label (68 articles, 43%). This is followed by the labels representing mixed types (not just continent, continental region, race) (24, 15%). The labels in the “Other” category were mostly *White British ancestry*, a label from the United Kingdom Biobank.

**TABLE 5:** Types of population labels employed by articles that use different types of data to operationalize ancestry. Because continental and continental region categories are those geographical categories most likely to be conflated with racial categories, we separate out those articles that used a mixed set of labels into those that represented a mixture between these types of label (continent, continental region, and race) from other types of mixtures.

Population labels used in the operationalization of ancestry	Type(s) of data used to operationalize ancestry N (%)				
	Genetic	Genetic and non-genetic	Non-genetic	Not specified	Total
Continent	29 (48)	4 (36)	15 (36)	24 (56)	72 (46)
Continental region	2 (3)	1 (9)	4 (10)	1 (2)	8 (5)
Country		1 (9)	3 (7)		4 (3)
Ethnicity	1 (2)		6 (14)	3 (7)	10 (6)
Mixed: just continent, continental region, race	9 (15)	2 (18)	1 (2)	6 (14)	17 (11)
Mixed: not just continent, continental region, race	7 (11)		7 (17)	7 (16)	21 (15)
No labels used	12 (20)	1 (9)		1 (2)	14 (9)
Others	1 (2)	2 (18)	3 (7)	1 (2)	7 (4)
Race			3 (7)		3 (2)
Total	61 (100)	11 (100)	42 (100)	43 (100)	157 (100)

A combined analysis of population labels and type of data used to operationalize ancestry (see Table 5) reveals that no matter what type of data is used (and when it was not clear what type of data was used), continental ancestry categories are the most common. This usage was highest (56%) when no indication is given of what type of data is used to operationalize ancestry. For all data types, most other types of labels besides continental are also used.

Among the articles which use ancestry categories, most types of population labels are used by articles from all the fields of study (see Supplementary Table S6). Continental ancestry is the most used population label in biology (23 articles, 47%) medicine (28, 51%), and public health (15, 48%). In sociology, ethnicity was the most popular label type (3, 38%). Anthropology was marked by a spread of different label types.

An analysis of how population labels are used across the authors’ country/region of institutional affiliation (see Supplementary Table S7) indicates that for those based everywhere save Asia, Continental ancestry labels are the most commonly used. In Asia, researchers most often used Mixed labels—a combination of continent and a country is typical (e.g., European and Japanese).

We observed several instances of grand generalizations from samples from populations with ethnic or country labels to continental groups, for example from Japanese ancestry to Asian ancestry (Brhane et al., 2020), and from French ancestry to European ancestry (Tonon et al., 2019).

## Discussion

In this discussion we first summarize our main findings, and then reflect on the normative consequences of our findings for the type of research we included in this empirical work. We start by noting that our results are limited by the types of articles, and subsequently, researchers, that we included. There are some types of research questions, namely those that do not have even a tangential relationship to human health, that we do not cover.

Our results indicate that ancestry is a concept that is drawn upon in multiple key ways across a broad range of types of research. This is particularly true when it comes to understanding traits and outcomes, from blood pressure to income. For researchers seeking to identify genetic variation linked to these traits, ancestry is evoked as a central part of the methodology, as something to be controlled for. For researchers interested in understanding social outcomes who are not interested in identifying genetic variants, ancestry is again something to be controlled for. For other researchers, ancestry shapes their research question; they hope that studying how a trait varies with ancestry can enable the identification of genetic contributions to between-group differences, including health disparities. Having established that researchers evoke ancestry in key ways, understanding what the concept means to them becomes central.

We demonstrate a huge diversity of understandings of ancestry amongst researchers. Several observations stand out. First, the concept of ancestry encompasses much more than genetics. Many of our interviewees, including geneticists, gave definitions that were broader than what could be inferred from genetic data, stressing for example the narrative aspect of ancestry. The example of “indigenous ancestry” also illustrates that ancestry is more than genetics; several indigenous groups have explicitly rejected genetics as relevant to questions of ancestry, in favor of cultural affiliation (TallBear 2013). Attempting to secure the term “ancestry” to refer to genetic variation—as done in e.g., (Mersha and Tilahun, 2015)—is not a viable strategy. Second, there is an absence of agreement on what is core to the concept of ancestry, for example, whether it has anything to do with geography. Third, many researchers, who were selected to be part of our sample precisely because their work engaged closely with the concept of ancestry, struggled to define it. Fourth, while some researchers stressed that ancestry was fundamentally different from the social constructs of race and ethnicity, we observed both in articles and in interviews frequent slippage between ancestry, race and/or ethnicity. Our results, which highlight the ways in which ancestry is ambiguous, can be read in the light of the broader literature on ambiguity in scientific concepts (reviewed in (Panofsky and Bliss 2017)). Some of this literature highlights positive roles that ambiguity can play, while the majority of the literature highlights negative functions of ambiguity, and the advantages that flow from standardization, see for example (Timmermans and Epstein 2010).

We also report a huge diversity of operationalizations of the concept of ancestry. The process of operationalization involves taking a definition of an abstract concept and making it measurable. Given the diversity of definitions of ancestry, it is perhaps not surprising there are so many operationalizations. The fundamental ambiguity of the concept, as discussed above, is also evidenced by our result that a quarter of all papers that operationalize ancestry fail to state anything about how this was done, for example whether inclusion criteria were based on self-reported information, geography, or genetics. This was particularly the case in Biology and Medicine, with Anthropology and Sociology articles more frequently stating what type of data was used to operationalize ancestry, perhaps reflecting greater sensitivity in these fields to the ways in which these categories reflect decisions made by researchers. The failure to specify what type of data is used to operationalize ancestry allows for the introduction of further ambiguity between genetics and social identities, particularly given that researchers often use “ancestry” language (rather than “genetic ancestry”) when genetic data has been used to operationalize the concept, and particularly when ancestry is operationalized more than once in the same article, sometimes using genetic data and sometimes not.

Our results also highlight that practices associated with using genetic data to operationalize ancestry rest on unclear conventions, and that there is a lack of clarity concerning the relationships between the different key concepts. This is particularly true for the use of Principal Components. PCs are sometimes referred to as ancestry, genetic ancestry, population structure, or some more qualified term, suggesting that they “capture” or “correlate” with one of the above. Researchers often justified their choices about the use of PCs by reference to prior papers, without being able to give their own justifications of why those choices were appropriate. This may be concerning, given the growing usage of PCs not only within statistical genetics but by those seeking “off the shelf” solutions to “control for genetics” (Boardman et al., 2010). It should also be concerning given that results can depend critically on choices made (Elhaik 2022).

The predominance of continental ancestry labels, particularly when genetic data is used and when it is not specified what type of data is used, reinforces concerns that the turn to genetic ancestry may just essentialize the quasi-racial groupings represented by these categories (Bliss 2020b; Lewis et al., 2022). On the other hand, the diversity of population labels in use helps demonstrate that researchers have a wide range of choices when it comes both to their operationalization of ancestry, and to their framing of their results.

In repeated guidelines for the use of population descriptors, transparency of what is meant by the concepts and how they are operationalized is presented as a minimum bar (Mauro et al., 2022). Our results show that, in the case of ancestry specifically, current research is very far from meeting this bar. Our results also help indicate why the goal of transparency may be hard to achieve: there is a huge diversity of ideas underlying the concept, which leads to a deep-running ambiguity about where the concept sits in relation to biology on the one hand and social identities on the other. The existing empirical work discussed in the background demonstrates the close links between racial ways of thinking and the way genetic ancestry is being conceptualized. By focusing our work not just on genetic ancestry, but on ancestry more broadly, we demonstrate additional ways in which this conflation happens.

The conflation between genetic and social ways of conceptualizing human difference—aided by the highly ambiguous term “ancestry”—is problematic because the concepts are importantly different (Cerdeña, Grubbs, and Non 2022). As Ian Hacking has pointed out, whereas there is a sense in which all concepts are socially constructed, some concepts are “interactive kinds,” in which the entities being classified know they are being classified, and act and are treated differently based on the ways that they are classified (Hacking 2001). People learn to treat people categorized by a structural system differently, depending on their category. This is true of race, which is a construct invented by white Europeans to secure their racial privilege (Omi and Howardt 2015). Genetic ancestry refers to how DNA is passed down through the human family tree (Mathieson and Scally 2020; Lewis et al., 2022). This human family tree has a complex structure; it is after all shaped by everything that shapes who has children with whom, which includes amongst other things geography and cultural practices. There are of course correlations between race and ethnicity and patterns of genetic variation, including at the continental level. But genetic variation is continuous, not categorical and not best represented by continental categories (Lewis et al., 2022)*.* And whereas racial labels are a result of sociopolitical processes, it is researchers who choose to impose categories on genetic data, and then to attach labels to those categories. As Bonham et al. write, “It is critical to avoid creating fictitious, discrete genomic groups while recognizing that self-identified race and ethnicity are highly associated with genetic ancestry at the continental and population level” (Bonham, Green, and Pérez-Stable 2018).

The ambiguity that use of the term “ancestry” provides is almost certainly helpful to researchers in some ways (Panofsky and Bliss 2017). But it also confuses our attempts to gain genuine understanding of the dynamics and processes in the creation of health outcomes. The motivation for much genetic research is to contribute to a causal understanding of why health-related traits are distributed the way they are. Genetic ancestry cannot be a causal factor in such analysis, it can only act as a proxy for underlying genetic variants that are playing a causal role. Racism can play a causal role, through many mechanisms that are being elucidated (see e.g., (Krieger 2021)). Inadequate reflection of the relation between these systems for capturing human difference can lead to incorrect conclusions. For example, attempts to explain differences in health outcomes based on differences in genetic ancestry are confounded by racism (Boulter et al., 2015). To make progress in accurately understanding the distribution of health outcomes, the different roles of genetic variation and social and environmental factors must be carefully considered.

While we agree with the dozens of other commentators on the importance of researchers transparently describing exactly who was studied, how they were classified, and why, we advocate for the following additional considerations.

(1) Use of the term “ancestry” by itself should be avoided. Rather, this term should always be qualified, for example as “genealogical ancestry” or “genetic ancestry”.
(2) Use of the term “population” should be avoided. It has no agreed upon meaning and only serves to make something sound more scientific than it in fact is. This is particularly true in genetics research when the term is apt to be confused with the term in population genetics theory for a group of individuals who are mating at random. Use of the term “group” is to be preferred, because it correctly draws attention to the question “by virtue of what are these individuals being grouped together” and because it evokes less scientific authority.
(3) Operationalizations of genetic ancestry that reflect the continuous nature of genetic variation, such as PCA, should be encouraged wherever possible (Duello et al., 2021; Lewis et al., 2022).
(4) If genetic ancestry must be operationalized using categories, multiple sets of categories should be used, to reflect the fact that one can carve up the human family tree in multiple ways (Lewis et al., 2022).
(5) Genetically inferred continental ancestry categories should only be used if necessary (Panofsky and Bliss 2017; Lewis et al., 2022).
(6) Researchers need to make themselves familiar with the key limitations of the tools they use.

The hope of some is that ancestry represents the biological, objective counterpart to the social constructs of race and ethnicity, and that a turn to ancestry categories could help us get away from the bad science and damaging history of previous classification systems and practices. Our investigation of how ancestry is actually used indicates it is a far cry from the objective and straightforward concept hoped for. Nor is it a uniquely “biological construct”. Indeed, the concept is fundamentally ambiguous, and not more conceptually clear than race or ethnicity in practice. By just moving to a new term, there is a danger that research in this area fails to address the known issues in the uses of race and ethnicity as population descriptors. Part of the problem is precisely that some scientists are searching for a more objective term, thus treating this set of issues as purely semantic when in fact the problems run deeper: we need more careful attention to the different roles of genetic variants (which are not uniformly distributed) on the one hand and the myriad other contributors to health outcomes on the other. The move to “ancestry” just confuses the issue. Given the central role ancestry plays in genetics research and beyond, these deep-seated issues with how ancestry is conceptualized and operationalized should raise concern, and highlight the importance of strategies that will advance conceptual clarity.

## Data Availability

The raw data supporting the conclusion of this article will be made available by the authors, without undue reservation.
