# Supplementary material for: Ancestry: How researchers use it and what they mean by it
Source: Front Genet. 2023 Jan 23;14:1044555. doi: 10.3389/fgene.2023.1044555 (PMC9900027; doi:10.3389/fgene.2023.1044555)
Supplement: Supplementary file 1 [file DataSheet1.PDF]

Supplementary Information for “Ancestry: how researchers use it, and what they mean by it”

Article inclusion strategy

Search strings within the Web of Science user interface, performed in February 2021

```
ALL="ancestry" AND ALL="chronic kidney disease", 2019+
ALL="ancestry" AND ALL="covid", 2019+
ALL="ancestry" AND ALL="prostate cancer", 2019+
ALL="ancestry" AND ALL="schizophrenia", 2019+
ALL=("ancestry" AND "health") AND WC="Sociology" 2010+
ALL=("ancestry" AND "health") AND WC="Anthropology" 2015+
ALL="ancestry" AND WC="Public, Environmental & Occupational Health" 2020+
```

The filtering of the articles returned by these searches was done by three coders (BD, SM, and AL) based on the abstract of the articles. Any fringe cases were marked as “maybe” for further review by the project team. Articles where the primary phenotype was a pathogenic reaction to a drug prescribed as treatment for the chosen disease were retained. Three COVID papers that discussed the ancestral lineages of SARS-CoV-2 and did not describe human ancestry were removed from the analysis.

Defining and assigning subfields

A list of subfields was defined by SM based on reading about the more general field, looking up different focuses/tracks in training programs, and sub-sections of specialization in larger professional associations. Both a primary and secondary subfield were selected by SM from the list of subfields by triangulating the methodology and design based on the abstract, the audience of the journal, and the affiliations of the first and last authors. To elaborate from closely related fields with an example: a paper was assigned “Medical Genetics” if it dealt with familial patterns of inheritance of specific traits, their testing, and their prevalence. A paper was assigned “Genetic Epidemiology” if it deployed a framework for identifying associations between genetic variation and a disease phenotype. But if it largely managed genomic data-sets to describe patterns of variation in one or more populations, it was assigned “Statistical Genetics.” Similar discernment was applied for distinguishing between the subfields of public health: “Community Health” and “Environmental Health.”

Establishing codebooks and initial coding

All documents — anonymized transcripts and articles — were uploaded to NVivo (release 1.6.1). For both sets of documents, codebooks were defined based on a mixture of an inductive and deductive process. First, codes were defined based on the research questions. A subset of documents were then coded by three coders (BD, SM, and AL), each of whom also identified new codes to capture excerpts that were interesting but did not fit into any of the existing codes. The results of this process were compared to both finalize a list of codes, and agree on how they should be applied. The same process was repeated again, with three coders coding a different set of documents. This process enabled us to establish inter-coder agreement. The articles were then coded by a single coder (BD), and the transcripts by two coders (AL and SM). The codebooks are given in Supplementary Tables 3 and 4.

To assign the type of data used to operationalize ancestry, the methodology, including any reference in the methodology to the Supplementary Information, was assessed for any indication of the data used. The word "ancestry" was also systematically searched for to attempt to identify how it was operationalized. Because some articles operationalized ancestry in more than one way, we chose to present this data on an operationalization-by-operationalization basis, rather than on an article-by-article basis. We grouped the types of data used for operationalization into genetic, non genetic, both, and not specified. This latter label was applied if ancestry categories were used in the analysis, or if the population was described in terms of ancestry, but it was impossible to tell what type of data was used for this operationalization. If the article did not operationalize ancestry, it was designated as "Not operationalized".

Based on the sections of text annotated with the "population label" code, we identified and listed all the population descriptors that were used to describe the participants per ancestry operationalization. We then used a population label grouping previously defined by Panofsky and Bliss (2017) to assign the descriptors into population labels, see Supplementary Table 5. We extend their "Race" category to include non US race-based designations. We also added the category "Mixed: Continent, continental region, race", and "Mixed: Other".

Qualitative analysis of coded sections

On a code-by-code basis we produced summary sentences of the coded sections, and used these to identify emergent themes. Results were discussed and refined with at least one other team member.

Supplementary Tables

Supplementary Table 1: The number of articles returned by each search, before and after filtering

Search	Before filter	In final set
Chronic kidney disease	54	20
COVID	18	10 (one overlap with Public Health)
Prostate cancer	75	52
Schizophrenia	68	37
Sociology	29	12
Anthropology	37	27
Public Health	52	48 (one overlap with COVID)

Supplementary Table 2: Subfields assigned to articles and interviewees

Subfield	Field	Definition (textbook)	Article Primary, N	Article Secondary, N	Interviewees, N
Environm	Public	Concerned with all aspects of the natural	16	6	1

ental Health	Health	and built environment affecting human health (e.g. toxicology).			
Community Health	Public Health	Focuses on people and their role as determinants of their own and other people's health.	22	12	1
Health Economics and Public Policy	Public Health	Study of the efficiency, effectiveness, value and behavior in the production and consumption of health and healthcare. Paired with study of the bureaucratic and legal structures of healthcare.	0	1	1
Health Services Administration	Public Health	Relating to leadership, management, and administration of public health systems, health care systems, hospitals, and hospital networks.	0	3	
Epidemiology	Public Health	The study and analysis of the distribution (who, when, and where), patterns and determinants of health and disease conditions in defined populations.	17	19	3
Oncology	Medicine	Deals with the prevention, diagnosis, and treatment of cancer.	27	14	2
Internal Medicine	Medicine	Dealing with the prevention, diagnosis, and treatment of internal diseases.	6	17	6
Pediatrics	Medicine	Involves the medical care of infants, children, and adolescents.	0	5	
Preventive Medicine	Medicine	Or prophylaxis, consists of measures taken for disease prevention.	2	1	
Neurology and Neuroscience	Medicine	Concerned with the structure, development, genetics, biochemistry, physiology, pharmacology and pathology of the nervous system.	2	5	
Medical Genetics	Medicine	Involves the diagnosis and management of hereditary disorders.	5	3	
Psychiatry	Medicine	Devoted to the diagnosis, prevention, study, and treatment of mental disorders.	23	14	3
Human Evolutionary Biology	Biology	Studies the evolutionary processes (natural selection, common descent, speciation)	3	16	4
Molecular Genetics	Biology	Concerned with describing how differences in the structures or expression of DNA molecules manifests as variation among organisms, the study of the mechanics of DNA.	2	5	1
Statistical Genetics	Biology	Concerned with the development of statistical methods for drawing inferences	6	7	2

		from genetic data (inclusive of Bioinformatics)			
Genetic Epidemiology	Biology	The study of the role of genetic factors in determining health and disease in families and in populations, and the interplay of such genetic factors with environmental factors.	36	39	5
Biochemistry	Biology	The study of chemical processes within and relating to living organisms.	0	1	1
Cell Biology	Biology	Concerned with the structure and function of the cell.	1	0	
Population Genetics	Biology	The study of genetic differences between populations, examining such phenomena as adaptation, speciation, and population structure.	6	5	1
Sociology of Science, Knowledge and Technology	Sociology	Or science, technology and society (STS); the study of how society, politics, and culture affect scientific research and technological innovation, and how these, in turn, affect society, politics and culture.	1	0	1
Medical Sociology	Sociology	Analysis of medical organizations and institutions; the production of knowledge and selection of methods, the actions and interactions of healthcare professionals, and the social or cultural effects of medical practice.	1	3	
Sociology of Stratification and Inequality	Sociology	Examines the distribution of wealth and power within and across societies and the systems of stratification that develop including class, caste, race, and gender.	1	6	2
Demography	Sociology	The study of statistics such as births, deaths, income, or the incidence of disease, which illustrate the changing structure of human populations.	1	3	
Social psychology	Sociology	Study of how the thoughts, feelings, and behaviors of individuals are influenced by the actual, imagined, and implied presence of Others; study of crowds and groups and their decisions	2	2	
Ethnic studies	Sociology	Interdisciplinary study of difference—chiefly race, ethnicity, and nation, but also sexuality, gender, and Others such markings—and power, as expressed by the state, by civil society, and by individuals.	5	10	3
Socio-Cul	Anthropol	The study of cultural variation among	0	1	1

tural Anthropol ogy	ogy	humans and the study of patterns of behavior.			
Physical Anthropol ogy	Anthropol ogy	Also biological anthropology; concerned with the biological and behavioral aspects of human beings, their extinct hominin ancestors, and related non-human primates, particularly from an evolutionary perspective	15	6	6
Archeolo gy	Anthropol ogy	The study of human activity through the recovery and analysis of artifacts, architecture, biological remains, sites, and cultural landscapes	5	1	

Supplementary Table 3: Codebook for systematic literature analysis

Code	Description
Admixture	Whenever admixture events, admixed populations, or admixed individuals are mentioned
Ancestry use case	Any process/practice of work for which the concept of ancestry is explicitly used (Note: often will overlap with population label use)
Explicit Ancestry Definition	If a paper gives an explicit definition of what it means by ancestry or genetic ancestry
Explicit ethical claim	Any ethical implications or adjudication given for using the named population in the study e.g. text relating to the potential for study results to stigmatize the research population or identification of the result of a population comparison result as morally problematic. Ethics approval for research is not counted as an ethical implications
Main research finding or Inference	Any instance or description that best delineates the main findings, implications or inferences from the research study e.g text that explains findings associating a gene loci with a trait
New finding or discovery claim	Explicit report of new findings or discoveries from study
Phenotype studied	Defined as the disease, condition or trait addressed in the research
Population clustering or grouping	Any instance or description that shows a pattern of grouping of populations due to the relative genetic similarity among human individuals and populations
Population comparison claim	Any between population observation, including if it includes generalization

Comparison with other studies	Findings are compared with previous results from other studies
Population definition	How the population studied is described/defined (when no subnode applies)
Datasets used	Either pre-existing data (e.g. thousands genomes) or details of how they assembled a dataset of genetic data
Exclusion criteria	Any instance of features or characteristics of the potential study participants that are adjudged unsuitable for selecting them for the study participation
Inclusion criteria	Any instance of features or characteristics of the potential study participants that qualify them suitable for selection to the study.
Sampling process	Any instance that describes the target population and how participants were selected for the research study.
Population generalization claim	How the study results are generalized
Population label use case	Process/practice of work for which population labels are used, e.g. sample accessioning
Explicit population category	The way the population in the articles is grouped. i.e. whether these are explicitly thought of as an ancestral, racial, or ethnic categories
Population labels	Every instance of a population label
Population partitioning justification	Any reasons given for dividing data into subpopulations, e.g. by race, for steps of the analysis
Population stratification or structure	Any mention of population stratification and/or population structure
Race or Ethnicity use case	Any instance, process of work for which the concepts of “ethnicity” or “race” are explicitly used (Note: may overlap with population label use or ancestry use case if there is conflation)
Research question, aim and objectives	Defined as the main aim and objective of the research
Study limitations	Any instance that describes the weaknesses in the research design and methodology that may influence outcomes and conclusions of the research.

Study motivation and justification	For example why the phenotype and why the population(s)
Suggestive future research	Any instance of text that describes the implications of the study findings that needs further research. It highlights aspects of the research that are salient to be studied in the future.
Theoretical claim about health disparities	This is a claim or discourse about population grouping and social factors that contribute to health disparities

Supplementary Table 4: Codebook for interview transcripts.

Code	Description
Admixture	Any mention of admixed individuals, methodologies for inferring admixture, “mixed race”, “mixed ethnicity”, etc.
Aspirations	The aspirations, hopes or desires for the future of their field
Communities	The importance of first hand knowledge of communities, and/or the importance of community engagement
Dataset	Any mention of specific databases that they used
Definition of ancestry	The explicit answer to the question “what does ancestry mean to you?” And any follow ups
Diversity or Minority populations	Any mention of “diversity”, both in datasets and in those who work in the field, and mention of “minority populations”
Evolution	Any mention of human evolutionary history, and in particular of natural selection
Fears or frustrations	Any discussions that have emotional connotation, or reflect the interviewee’s gripes with their work
Funding	The ways in which funders influence the work that gets done
Generalization or Comparision claim	From/between one group to another, for example when interviewees are describing health differences between groups, discussing the prevalence of a variant in specific populations, or when extrapolating based on something they believe about a group
Health disparities	Any mention of health disparities, for example in motivating work
History	Any discussion of historical events, such as migrations, population bottlenecks, marriage laws, etc, and other mention of the relevance of history for their work.
Interdisciplinarity	Mention of people from other disciplines who they worked with, and explicit calls for interdisciplinary work

Justification for choices	Including for choice of phenotype, choice of population and methodological strategies. often in response to questions about their paper and the direction of their research.
Personal connection	Any appeal to a personal story to illustrate a point
Population concepts	Anything about these concepts that is more than the use of a label. There are subnodes for Ancestry, Country, Ethnicity, Genetic ancestry, Indigeneity, Language, Race, Self-identification.
Population structure or stratification	Any explicit mention of these terms, or of "population substructure"
Publishing	Anything about the process of getting this or other work published, including referee comments
Racism or Discrimination	Any mention of the relevance of racism or other forms of discrimination, including both as experienced in science or as a broader cause of health outcomes
Sample size	Any explicit mentions of samples, their size, limitations.
Scale of categories	Any mention of decisions to split or group research participants into different categories.
Social determinants of health	Any mention of the relevance of the social determinants of health, sometimes discussed as environmental or structural determinants of health
Statistical methodology	Any discussion of the use of a statistical methodology. There are subnodes for Genome Wide Association Studies (GWAS), Polygenic Risk Scores (PRS), and Principal Component Analysis (PCA).
Status of the field	Any description of the way things are done or general remarks on the trajectory of their field or others
Use cases for ancestry	Any description of discrete practices of work, e.g. “To control for population stratification”, “To assess diversity”
Where they'd point someone	In response to the question asking where they would point a junior colleague interested in these issues

Supplementary Table 5: Types of population labels, with examples

**Population Label**	**Example of population descriptors**
Continent	European Ancestry, African Ancestry, Asian Ancestry
Continental region	Northern European, West African, East Asian
Country	Netherlands (or Dutch), Japanese
Ethnicity	Han, Gond
Race	Based on a logic of defining people in terms of biologically based group difference, including US Census (White or

	Caucasian, Black or African American, Hispanic or Latino), and similar schemes from other countries
Other	Typically a mixture of types, E.g. White British ancestry, Non-white Brazilian
Mixed: just continent, continental region, race	E.g. a set of: “African, Hispanic/Latino, European, and East Asian”
Mixed: not just continent, continental region, race	E.g. a set of: “European ancestry, Turkish ancestry”

Supplementary Table 6: For the articles that use ancestry categories, the population labels used in different fields of study

**Population labels**	**Primary Field**					
	Anthropology N (%)	Biology N(%)	Medicine N(%)	Public Health N(%)	Sociology N(%)	Total N (%)
Continent	5 (36)	23 (47)	28 (51)	15 (48)	1 (13)	72 (46)
Continental region	2 (14)	2 (4)	2 (4)	1 (3)	1 (13)	8 (5)
Country		2 (4)	1 (2)	1 (3)		4 (3)
Ethnicity	3 (21)	2 (4)	2 (4)		3 (38)	10 (6)
Mixed: Continent, continental region, race	1 (7)	7 (14)	8 (14)	2 (6)		18 (11)
Mixed: Other	2 (14)	7 (14)	4 (7)	6 (19)	2 (25)	21 (14)
No labels	1 (7)	4 (8)	7 (13)	2 (6)		14 (9)
Others		1 (2)	3 (5)	3 (10)		7 (4)
Race		1 (2)		1 (3)	1 (13)	3 (2)
**Total**	**14 (100)**	**49 (100)**	**55 (100)**	**31 (100)**	**8 (100)**	**157 (100)**

Supplementary Table 7: The Population Labels used for ancestry categories broken down by location of the primary affiliation of the first author of the articles

**Population**	**Country/Region of Authors Affiliation**

**labels**	Africa N (%)	Asia N(%)	Europe N(%)	Latin America N(%)	Others N(%)	United States of America N(%)	Total N(%)
Continent	2 (50)	2 (17)	15 (48)	4 (40)	10 (59)	39 (47)	72 (46)
Continental region			3 (10)		1 (6)	4 (5)	8 (5)
Country		1 (8)		1 (10)		2 (2)	4 (3)
Ethnicity		2 (17)	1 (3)	3 (30)	1 (6)	3 (4)	10 (6)
Mixed: Continent, continental region, race		1 (8)	3 (10)		1 (6)	13 (16)	18 (11)
Mixed: Other	1 (25)	5 (42)	4 (13)	1 (10)	2 (12)	8 (10)	21 (13)
No labels		1 (8)	2 (6)			11 (13)	14 (9)
Others	1 (25)		3 (10)	1 (10)	2 (12)		7 (4)
Race						3 (4)	3 (2)
**Total**	**4 (100)**	**12 (100)**	**31 (100)**	**10 (100)**	**17 (100)**	**83 (100)**	**157 (100)**
